# Effect of 12 months of testosterone replacement therapy on metabolic syndrome components in hypogonadal men: data from the Testim Registry in the US (TRiUS)

**DOI:** 10.1186/1472-6823-11-18

**Published:** 2011-11-01

**Authors:** Rajib K Bhattacharya, Mohit Khera, Gary Blick, Harvey Kushner, Dat Nguyen, Martin M Miner

**Affiliations:** 1 University of Kansas Medical Center, 3901 Rainbow Blvd, MS2024, Kansas City, KS 66160, USA; 2 Scott Department of Urology, Baylor College of Medicine, 6620 Main Street, Suite 1325, Houston, TX 77030, USA; 3 Circle Medical LLC, 153 East Avenue, Suite 32, Norwalk, CT 06851, USA; 4 Auxilium Pharmaceuticals, 40 Valley Stream Parkway, Malvern, PA 19355, USA; 5 Miriam Hospital Men's Health Center, 164 Summit Avenue, Providence, RI 02906, USA

**Keywords:** Testosterone, metabolic syndrome, obesity, testosterone gel, testosterone replacement, TRiUS registry, Testim, hypogonadism, testosterone deficiency, fasting glucose

## Abstract

**Background:**

Recent evidence suggests that there may be a bidirectional, physiological link between hypogonadism and metabolic syndrome (MetS), and testosterone replacement therapy (TRT) has been shown to improve some symptoms of MetS in small patient populations. We examined the effect of 12 months of TRT on MetS components in a large cohort of hypogonadal men.

**Methods:**

Data were obtained from TRiUS (Testim^® ^Registry in the United States), a 12-month, multicenter, prospective observational registry (N = 849) of hypogonadal men prescribed Testim 1% testosterone gel (5-10 g/day). Data analyzed included age, total testosterone (TT), free testosterone (FT), sex hormone-binding globulin (SHBG), and MetS components: waist circumference, blood pressure, fasting blood glucose, plasma triglycerides, and HDL cholesterol.

**Results:**

Of evaluable patients (581/849) at baseline, 37% were MetS+ (n = 213) and 63% were MetS- (n = 368). MetS+ patients had significantly lower TT (p < 0.0001) and SHBG (p = 0.01) levels. Patients with the lowest quartile TT levels (<206 ng/dL [<7.1 nmol/L]) had a significantly increased risk of MetS+ classification vs those with highest quartile TT levels (≥331 ng/dL [≥11.5 nmol/L]) (odds ratio 2.66; 95% CI, 1.60 to 4.43). After 12 months of TRT, TT levels significantly increased in all patients (p < 0.005). Despite having similar TT levels after TRT, only MetS+ patients demonstrated significant decreases in waist circumference, fasting blood glucose levels, and blood pressure; lowest TT quartile patients demonstrated significant decreases in waist circumference and fasting blood glucose. Neither HDL cholesterol nor triglyceride levels changed significantly in either patient population.

**Conclusion:**

Hypogonadal MetS+ patients were more likely than their MetS- counterparts to have lower baseline TT levels and present with more comorbid conditions. MetS+ patients and those in the lowest TT quartile showed improvement in some metabolic syndrome components after 12 months of TRT. While it is currently unclear if further cardiometabolic benefit can be seen with longer TRT use in this population, testing for low testosterone may be warranted in MetS+ men with hypogonadal symptoms.

## Background

Hypogonadism is commonly found in middle-aged and older men [1]; symptoms may include decreased muscle mass and strength, increased abdominal fat, decreased sexual interest and function, depressed mood, and fatigue [2]. While the prevalence and symptoms of hypogonadism are associated with aging [3], the presence of certain comorbidities and cardiovascular risk factors are also associated with low testosterone levels [1], including the components of metabolic syndrome (MetS) [4]. According to the ATP III and AHA/NHLBI guidelines [5,6] the components of MetS include: insulin resistance, increased waist circumference, high triglycerides, low high-density lipoprotein (HDL) cholesterol, and increased blood pressure (BP).

The worldwide prevalence of MetS has dramatically increased over the past 20 years [7]. Evidence suggests that there may be a bidirectional, physiological link between hypogonadism and MetS [4]. Reduced testosterone levels can lead to muscle loss and weight gain, and is a significant predictor of MetS and type 2 diabetes in men [8-10]. Hyperinsulinemia and obesity may inhibit testicular testosterone production [10]. Testosterone replacement therapy (TRT) in hypogonadal men improves lean body mass and reduces fat mass [11], and may improve some symptoms of MetS and type 2 diabetes [12-14]. However, there have been four placebo-controlled, randomized trials [15-18] and a meta-analysis [19] published to date that have effectively evaluated the potential of TRT for metabolic symptom improvement.

There is a lack of studies of general populations of hypogonadal men that examined the effect of TRT on metabolic symptoms. We used data collected through the Testim^® ^Registry in the United States (TRiUS) to investigate the prevalence of MetS and associated comorbidities in a large community-based population of men with hypogonadism and examined the effects of long-term (12-month) TRT on MetS components.

TRiUS was established as a prospective observational study (ie, patient registry) of hypogonadal men (N = 849) treated with testosterone gel (Testim 1%). The objective of this analysis was to determine whether restoring testosterone levels in men with hypogonadism in typical clinical settings could improve components of MetS.

## Methods

Data were part of the TRiUS patient registry of hypogonadal men, whose methodology and baseline demographics have been described previously [20]. As with other patient registry or observational study designs, the TRiUS methodology reflected typical clinical practice: there was no placebo control or control patient population and data collection was limited to the patients who were available at each time point. Inclusion criteria for the registry included hypogonadal males who provided informed consent and were naïve to Testim therapy at enrollment, though not necessarily naïve to other forms of TRT. Exclusion criteria for the registry included patients who demonstrated hypersensitivity to any ingredients in the testosterone gel (including testosterone), had breast cancer, or had known or suspected prostate cancer. All study sites had local or central Institutional Review Board (IRB) approval, and research was carried out in compliance with the Declaration of Helsinki as currently amended.

Testosterone gel dosing was prescribed at the physician's discretion and could be titrated up or down at any time during the 12-month study period in order to achieve the desired steady-state total testosterone (TT) level. Follow-up examinations were suggested at approximately 1, 3, 6, and 12 months after the initiation of therapy, although actual follow-up visits were also at the discretion of the physician. Visits occurring within a specified time window of a suggested time point (±14 days for month 1, ±30 days for months 3 and 6, and visits at day 330 or later for month 12) were grouped within that time point. If a patient had multiple visits within the specified time window before or after a time point, the visit closest to the designated time point was used.

Suggested assessments at visits included, but were not limited to: testosterone levels (total, free, and bioavailable), PSA, anthropometric measures, vital signs, cholesterol panel, hematology, and fasting glucose measures, and were measured according to the physician's usual practice, including the measurement of free testosterone. Patient self-report of comorbid conditions and diseases such as diabetes, hypertension, dyslipidemia, and sleep apnea were recorded, as well as use of antidepressants, opioids, and phosphodiesterase type 5 (PDE5) inhibitors.

Assessment measures and medical history from electronic clinical response forms were used to characterize men as positive (MetS+) or negative (MetS-) for MetS. ATP III and AHA/NHLBI criteria [5,6] required 3 of 5 of the following characteristics for MetS+ classification: central obesity (waist circumference >102 cm [40 inches]), high BP (systolic BP ≥130 mmHg or diastolic BP ≥85 mmHg or medical history of hypertension), high fasting blood glucose (>100 mg/dL [>5.6 mmol/L]), hyperlipidemia (triglycerides >150 mg/dL [>1.7 mmol/L]), and low HDL cholesterol (<40 mg/dL [<1.0 mmol/L]).

If 3 of 5 criteria could not give a definitive MetS+ or MetS- classification, then the patient was excluded from analysis. For example, if a patient was positive for 2 of 4 criteria but data on the fifth criteria were missing, then no classification could be made and the patient was excluded from further analyses (ie, was not included in either group). Data for all 5 criteria were not necessary for inclusion if available data could give a definitive MetS classification.

### Statistical analysis

Comparisons between subjects classified as MetS+ vs MetS- were performed using Fisher's exact test for categorical variables, and t-tests of means for numerically continuous variables. Baseline correlations were examined using the Pearson (r) first-order partial correlation coefficient adjusting for age. The continuous effect of age on testosterone levels at baseline was examined using an analysis of covariance (ANCOVA). Odds ratios were calculated using logistic regression models. All changes from baseline values were examined using repeated measures analysis of variance (ANOVA) based on a mixed model with two fixed effects: Post-baseline visit (months 3, 6, and 12) and either MetS status (+ or -) or baseline TT quartile. P-values ≤0.05 were considered statistically significant and no Bonferroni corrections were made for multiple comparisons. All analyses were performed using an a priori analysis plan with SAS^® ^(SAS Institute, Cary NC, version 9.1).

## Results

MetS criteria were examined for the 849 patients enrolled in the TRiUS registry. At baseline, 32% (268/849) did not have enough data to determine MetS status and were excluded from this analysis. Of those evaluated (n = 581), 213 (37%) patients were classified as with MetS (MetS+), and 368 (63%) patients were classified as without (MetS-). The two cohorts were statistically different (p = 0.04) in mean age (Table 1).

**Table 1:** Baseline characteristics

Characteristic	Overall (N = 849)	MetS+ (n = 213)	MetS- (n = 368)	p-Value (+ vs -)
		**Mean ± SD**		

Mean age, yrs (range)	52.1 ± 12.3 (20-85) (n = 845)	53.0 ± 11.3 (21-83) (n = 212)	50.9 ± 12.2 (24-85) (n = 367)	0.04

Hip circumference, cm [inches]	106.4 ± 14.7 [41.9 ± 5.8] (n = 836)	114.0 ± 15.0 [44.9 ± 5.9] (n = 212)	100.1 ± 12.2 [39.4 ± 4.8] (n = 364)	<0.0001

BMI, kg/m^2^	31.4 ± 6.9 (n = 846)	34.6 ± 6.6 (n = 213)	28.7 ± 6.4 (n = 368)	<0.0001

Waist circumference, cm [inches]	103.9 ± 16.5 [40.9 ± 6.5] (n = 835)	114.3 ± 16.0 [45.0 ± 6.3] (n = 211)	95.5 ± 13.7 [37.6 ± 5.4] (n = 363)	<0.0001

Fasting blood glucose, mg/dL [mmol/L]	106.4 ± 36.6 [5.9 ± 2.0] (n = 455)	118.3 ± 40.0 [6.6 ± 2.2] (n = 189)	96.6 ± 31.2 [5.4 ± 1.7] (n = 244)	<0.0001

Systolic BP, mmHg	129.2 ± 15.3 (n = 845)	134.3 ± 15.8 (n = 213)	122.8 ± 12.9 (n = 364)	<0.0001

Diastolic BP, mmHg	79.5 ± 9.5 (n = 845)	82.5 ± 9.6 (n = 213)	76.1 ± 8.3 (n = 365)	<0.0001

HDL cholesterol, mg/dL [mmol/L]	43.3 ± 12.6 [1.1 ± 0.3] (n = 468)	38.1 ± 9.6 [1.0 ± 0.2] (n = 203)	47.8 ± 12.8 [1.3 ± 0.3] (n = 241)	<0.0001

LDL cholesterol, mg/dL [mmol/L]	104.3 ± 37.9 [2.7 ± 1.0] (n = 456)	99.7 ± 36.1 [2.6 ± 0.9] (n = 196)	107.3 ± 37.7 [2.8 ± 1.0] (n = 235)	0.04

Total cholesterol, mg/dL [mmol/L]	182.7 ± 45.4 [4.7 ± 1.2] (n = 472)	179.7 ± 45.5 [4.66 ± 1.2] (n = 205)	183.4 ± 43.5 [4.8 ± 1.1] (n = 241)	0.4

Plasma triglycerides, mg/dL [mmol/L]	175.8 ± 121.1 [2.0 ± 1.4] (n = 474)	212.1 ± 134.3 [2.4 ± 1.5] (n = 208)	141.4 ± 94.9 [1.6 ± 1.1] (n = 239)	<0.0001

Abbreviations: BMI = body mass index; BP = blood pressure; HDL = high-density lipoprotein; LDL = low-density lipoprotein; MetS = metabolic syndrome.

Of the 581 evaluable patients at baseline, a variable number were available for data collection at 3, 6, and 12 months of TRT: 160 patients at 3 months, 233 patients at 6 months, and 218 patients at 12 months. The percentages of MetS+ patients were as follows: 41.3% (66/160) at 3 months, 36.9% (86/233) at 6 months, and 40.4% (88/218) at 12 months. The remaining percentages were MetS- patients.

### Analysis at baseline

#### Comparison between MetS+ and MetS- patients at baseline

**Figure 1:**
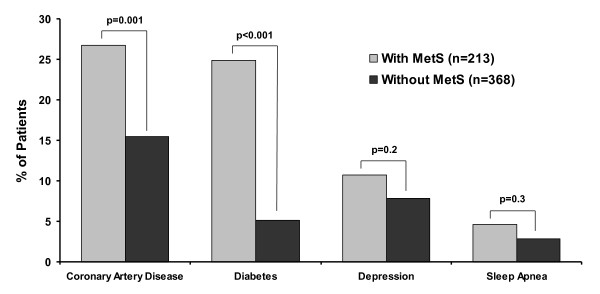
**Percentage of patients with comorbid conditions at baseline, by MetS status**. As expected, the percentage of patients with cardiovascular disease and diabetes was statistically higher in the MetS+ cohort (coronary artery disease [p = 0.001], and diabetes [p < 0.001]). Abbreviation: MetS = metabolic syndrome.

As expected, MetS+ and MetS- cohorts showed statistically significant differences in MetS components and related measures (Table 1) and in the prevalence of cardiovascular and metabolic comorbid conditions (Figure 1).

#### Odds ratios of MetS status by TT and SHBG levels at baseline

**Figure 2:**
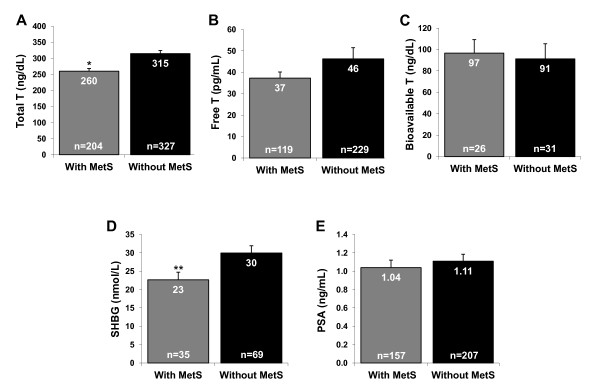
**Baseline testosterone, SHBG, and PSA**. Baseline values (mean ± SE) for total testosterone (A), free testosterone (B), bioavailable testosterone (C), SHBG (D), and PSA (E) in patients with and without MetS. Total serum testosterone and SHBG levels were statistically lower in the MetS+ group. *p < 0.0001, **p = 0.01. Abbreviations: MetS = metabolic syndrome; PSA = prostate-specific antigen; SHBG = sex hormone-binding globulin; T = testosterone.

Because MetS+ patients had significantly lower TT and SHBG levels (Figure 2), we examined if either of these levels were associated with an increased chance of being MetS+. To study the effect of TT levels, those patients with baseline TT measures (n = 528) were divided into quartiles based upon the distribution of testosterone levels among this population: <206, 206-256, 257-330, and ≥331 ng/dL [<7.1, 7.1-8.9, 8.9-11.5, and ≥11.5 nmol/L]. The percentage of MetS+ patients in each quartile were as follows: Quartile 1 (<206 ng/dL [<7.1 nmol/L]) = 50.4% (66/131); Quartile 2 (206-256 ng/dL [7.1-8.9 nmol/L]) = 39.1% (50/128); Quartile 3 (257-330 ng/dL [8.9-11.5 nmol/L]) = 37.8% (51/135); and Quartile 4 (≥331 ng/dL [≥11.5 nmol/L]) = 27.6% (37/134). As expected, the highest percentage of MetS+ patients was in the lowest TT quartile.

Using the highest quartile (≥331 ng/dL [≥11.5 nmol/L]) as a reference, the odds ratios for MetS+ status among TT level quartiles were as follows: Quartile 1 (<206 ng/dL [<7.1 nmol/L]) = 2.66 (95% CI, 1.60 to 4.43); Quartile 2 (206-256 ng/dL [7.1-8.9 nmol/L]) = 1.68 (95% CI, 1.00 to 2.82); and Quartile 3 (257-330 ng/dL [8.9-11.5 nmol/L]) = 1.59 (95% CI, 0.95 to 2.66). The odds of being classified as MetS+ were significantly greater (p = 0.002) for patients in Quartile 1 (TT <206 ng/dL [<7.1 nmol/L]) than those in Quartile 3 (TT 257-330 ng/dL [8.9-11.5 nmol/L]) in a hypogonadal population.

SHBG levels (n = 104) were divided by the median value (≤23 and >23 nmol/L). The percentages of MetS+ patients were 39.1% in the low SHBG (≤23 nmol/L) group and 29.3% in the normal SHBG (>23 nmol/L) group. The odds ratio of being classified as MetS+ for the low SHBG group vs the normal SHBG group was 1.55 (95% CI, 0.68 to 3.52) and was not significant (p = 0.3). Thus, TT levels seemed to be a better indicator of metabolic syndrome status than SHBG levels in our study.

#### Correlations of baseline TT levels with anthropometric and blood pressure measures

These odds ratios suggested that TT levels may correlate to the components of MetS. We found that TT levels significantly and inversely correlated (p < 0.005) with all anthropometric and hemodynamic measures (Table 2), even after partialing out the effects of age. Free testosterone, bioavailable testosterone, and SHBG levels did not significantly correlate with MetS components, with the exception of free testosterone and diastolic blood pressure (p = 0.04).

**Table 2:** Baseline Pearson correlation coefficients after adjusting for age

	Total Testosterone	Free Testosterone	Bioavailable Testosterone	SHBG
	**Pearson r, p-Value (n)**			

Waist circumference	-0.20, <0.0001 (525)	0.01, 0.8 (340)	-0.14, 0.3 (55)	-0.10, 0.3 (102)

Hip circumference	-0.15, 0.0005 (527)	0.02, 0.7 (341)	-0.19, 0.2 (55)	-0.10, 0.3 (102)

Weight	-0.19, <0.0001 (531)	0.04, 0.4 (344)	-0.02, 0.9 (56)	-0.17, 0.09 (104)

BMI	-0.22, <0.0001 (531)	0.04, 0.4 (344)	-0.14, 0.3 (56)	-0.18, 0.07 (104)

SBP	-0.12, 0.005 (528)	0.04, 0.5 (341)	-0.15, 0.3 (56)	0.05, 0.6 (104)

DBP	-0.20, <0.0001 (529)	-0.11, 0.04 (342)	-0.08, 0.6 (56)	0.0003, 1.0 (103)

Abbreviations: BMI = body mass index; DBP = diastolic blood pressure; SBP = systolic blood pressure; SHBG = sex hormone-binding globulin.

### Analysis after testosterone replacement therapy

We analyzed data from the 3-, 6-, and 12-month follow-up records of the 581 evaluable MetS+ and MetS- registry patients. Prescription compliance (calculated for each patient as total number of compliant days divided by the total number of days prescribed TRT) was based on patient diary information and could be determined in 55% (321/581) of the population; compliance rates were comparable between MetS+ and MetS- patients (90.7 ± 1.5%, n = 107 vs 93.6 ± 0.9%, n = 189, respectively; p = 0.1).

#### MetS component responses to TRT by metabolic syndrome status and TT level

TT significantly increased from baseline values in both MetS+ and MetS- patients at all time points during TRT (Table 3); however, free testosterone did not significantly change from baseline in MetS- patients after 6 and 12 months TRT and SHBG levels did not change in either cohort at any time point. Neither mean TT levels nor changes in TT levels from baseline were significantly different between MetS+ and MetS- patients after 3, 6, and 12 months of TRT (Table 3).

**Table 3:** TT, FT, and SHBG change from baseline after 3, 6, and 12 months of TRT

	MetS+	MetS-	p-Value, Differences MetS+ vs MetS-	
	**Mean Value, Mean Change from Baseline** **(p-Value for Change from Baseline)**		**Mean Value**	**Mean Change From Baseline**

**TT, ng/dL [nmol/L]**				

3 Months	505 [17.5], +223 (<0.0001)	503 [17.5], +197 (<0.0001)	0.9	0.7

6 Months	398 [13.8], +172 (<0.0001)	455 [15.8], +123 (0.0002)	0.09	0.3

12 Months	487, +261 (<0.0001)	512, +190 (<0.0001)	0.5	0.2

**FT, pg/mL [pmol/L]**				

3 Months	84.1 [291.8], +36.8 (0.02)	93.8 [325.5], +39.6 (0.003)	0.6	0.9

6 Months	77.4 [268.6], +55.8 (0.0001)	63.5 [220.3], +12.6 (0.2)	0.3	0.01

12 Months	77.5 [269.0], +49.7 (0.0007)	68.8 [238.7], +15.0 (0.2)	0.5	0.05

**SHBG, nmol/L**				

3 Months	24.6, +1.67 (0.7)	31.7, -0.06 (1.0)	0.1	0.7

6 Months	24.3, -1.37 (0.7)	31.2, -1.64 (0.6)	0.1	1.0

12 Months	17.7, -6.93 (0.1)	33.4, -0.80 (0.8)	0.005	0.3

Abbreviations: FT = free testosterone; SHBG = sex hormone-binding globulin; TT = total testosterone.

**Figure 3:**
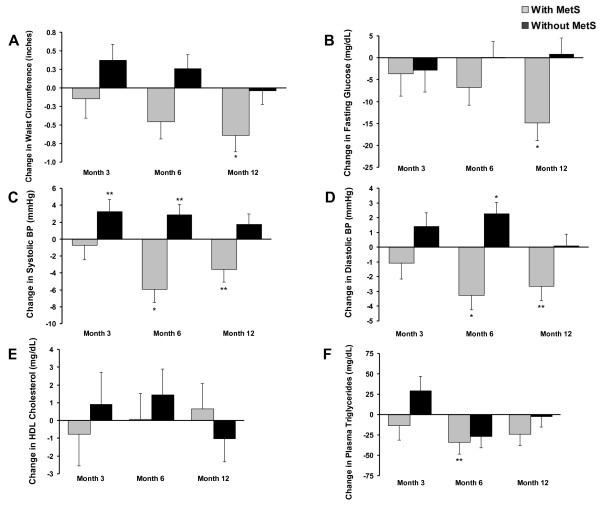
**Change from baseline in MetS components**. Change from baseline (mean ± SE) in waist circumference (A), fasting blood glucose (B), systolic BP (C), diastolic BP (D), HDL cholesterol (E), and plasma triglycerides (F) in patients with metabolic syndrome (gray) and without metabolic syndrome (black) at 3, 6, and 12 months on TRT. *p ≤ 0.005, **p ≤ 0.05. Abbreviations: BP = blood pressure; HDL = high-density lipoprotein; MetS = metabolic syndrome; TRT = testosterone replacement therapy.

Despite achieving similar TT levels after TRT, treatment had different effects on MetS components in MetS+ and MetS- patients (Figure 3). Statistically significant improvements in waist circumference, fasting blood glucose, and blood pressure were found in MetS+ patients after 12 months of TRT. MetS- patients did not show improvement in any metabolic syndrome criteria at any time point. It is unclear if these outcomes were related to TRT dose since patients could be titrated up or down during treatment as determined by the physician to achieve steady TT levels.

Because we found that patients in the lowest TT quartile at baseline were at an increased risk for metabolic syndrome classification, we examined if changes in MetS components after TRT were different for patients in the different quartiles. We found that TT levels significantly increased from baseline at all time points in patients in the lowest three quartiles (Quartiles 1-3), but not in the highest quartile (Quartile 4). The difference among the groups in change from baseline was significant (Table 4). However, the mean TT levels achieved at months 3, 6, and 12 of TRT were not significantly different among the groups; thus, the quartiles differed in change in TT, but not in mean TT levels, during TRT. In contrast, SHBG levels did not significantly change from baseline in any quartile at any time point. However, the difference between mean SHBG levels among the quartiles after 12 months of TRT was significant (p = 0.002). Lastly, significant changes in free testosterone were variable across quartiles and over time within quartiles; unlike TT, the quartiles did not significantly differ from each other in mean free testosterone levels, nor in change from baseline.

**Table 4:** TT, FT, and SHBG change from baseline after 3, 6, and 12 months of TRT, by baseline testosterone level quartiles

	Quartile 1 (<206 ng/dL) [<7.1 nmol/L]	Quartile 2 (206-256 ng/dL) [7.1-8.9 nmol/L]	Quartile 3 (257-330 ng/dL) [8.9-11.5 nmol/L]	Quartile 4 (≥331 ng/dL) [≥11.5 nmol/L]	p-Value, Differences Among Quartiles	
	**Mean Value, Mean Change from Baseline** **(p-Value for Change from Baseline)**				**Mean Value**	**Mean Change From Baseline**

**TT, ng/dL [nmol/L]**						

3 Months	465 [16.1], +307 (<0.0001)	466 [16.2], +234 (<0.0001)	543 [18.8], +248 (0.0002)	520 [18.0], +59 (0.3)	0.7	0.02

6 Months	405 [14.1], +244 (<0.0001)	379 [13.2], +148 (0.0007)	425 [14.7], +135 (0.01)	535 [18.6], -3.7 (0.9)	0.09	0.002

12 Months	519 [18.0], +359 (<0.0001)	456 [15.8], +225 (<0.0001)	496 [17.2], +206 (0.0003)	520 [18.0], +3.6 (0.9)	0.7	<0.0001

**FT, pg/mL [pmol/L]**						

3 Months	74 [256.8], +32 (0.1)	93 [322.7], +19 (0.5)	121 [419.9], +80 (0.0002)	80 [277.6], +17 (0.03)	0.3	0.1

6 Months	84 [291.5], +57 (0.0007)	71 [246.4], +3.8 (0.8)	82 [284.5], +45 (0.02)	69 [239.4], +6.3 (0.7)	0.8	0.06

12 Months	87 [301.9], +59 (0.0008)	67 [232.5], +9.0 (0.7)	56 [194.3], +18 (0.3)	70 [242.9], +6.9 (0.7)	0.5	0.1

**SHBG, nmol/L**						

3 Months	27.9, +2.83 (0.7)	24.1, +0.1 (1.0)	32.1, +1.52 (0.7)	37.2, -1.66 (0.7)	0.2	0.9

6 Months	25.6, -1.00 (0.9)	25.7, +3.30 (0.6)	26.5, -3.84 (0.4)	38.9, -1.49 (0.7)	0.06	0.8

12 Months	19.4, -6.50 (0.4)	20.7, -1.45 (0.8)	25.2, -6.72 (0.1)	45.8, +5.75 (0.3)	0.002	0.3

Abbreviations: FT = free testosterone; SHBG = sex hormone-binding globulin; TT = total testosterone.

For each TT quartile, we examined changes in metabolic syndrome components after 12 months of TRT (Table 5). The only significant changes, found in Quartile 1 (<206 ng/dL [<7.1 nmol/L]), were for a mean loss of 2.1 cm [0.84 inches] in waist circumference (p = 0.002) and a drop of 11.5 mg/dL (0.64 mmol/L) in fasting glucose levels (p = 0.01). Nevertheless, there was no significant difference between the quartiles in metabolic syndrome components.

**Table 5:** 12-month changes from baseline in MetS components and cardiovascular values by baseline testosterone level quartiles

	Quartile 1 (<206 ng/dL) [<7.1 nmol/L]	Quartile 2 (206-256 ng/dL) [7.1-8.9 nmol/L]	Quartile 3 (257-330 ng/dL) [8.9-11.5 nmol/L]	Quartile 4 (≥331 ng/dL) [≥11.5 nmol/L]	Differences Among Quartiles
	**Mean Change from Baseline** **(p-Value)**				**p-Value**

Waist circumference, cm [inches]	-2.1 [-0.84] (0.002)	-1.2 [-0.49] (0.1)	+0.5 [+0.20] (0.5)	-0.1 [-0.03] (0.9)	0.06

FBG, mg/dL [mmol/L]	-11.5 [-0.64] (0.01)	+0.76 [+0.04] (0.9)	-7.7 [-0.43] (0.1)	+1.1 [+0.06] (0.8)	0.2

Systolic BP, mmHg	-1.5 (0.4)	-1.0 (0.6)	-2.9 (0.2)	+1.9 (0.4)	0.4

Diastolic BP, mmHg	-1.5 (0.2)	-2.3 (0.07)	-2.5 (0.09)	+0.3 (0.8)	0.4

HDL cholesterol, mg/dL [mmol/L]	-0.45 [-0.01] (0.8)	-2.1 [-0.05] (0.3)	-0.89 [-0.02] (0.6)	+0.54 [+0.01] (0.8)	0.8

Plasma TG, mg/dL [mmol/L]	-17.8 [-0.20] (0.3)	-11.4 [-0.12] (0.6)	-34.2 [-0.39] (0.08)	+2.1 [+0.02] (0.9)	0.7

BP = blood pressure; FBG = fasting blood glucose; HDL = high-density lipoprotein; MetS = metabolic syndrome; TG = triglycerides.

#### Correlations of TT with MetS components after TRT

In the entire patient population, we examined the correlations of final TT levels with MetS components and related measures. At 12 months, laboratory values and anthropometrics were measured in 214 of 218 returning patients and TT levels were measured in 117 of these patients. At this final time point, TT levels significantly and inversely correlated with anthropometric measures and glucose levels, even after partialing out the effects of age: waist circumference (r = -0.37, p < 0.001, n = 115), body mass index (BMI) (r = -0.34, p < 0.001, n = 115), weight (r = -0.28, p = 0.003, n = 113), hip circumference (r = -0.31, p < 0.001, n = 115) and fasting blood glucose (r = -0.26, p = 0.03, n = 74). TT levels did not correlate with systolic or diastolic BP, HDL cholesterol, or plasma triglycerides.

In addition, the change in TT levels over 12 months TRT significantly and inversely correlated with the changes in weight (r = -0.23, p = 0.02, n = 109) and BMI (r = -0.23, p = 0.02, n = 109), but not with fasting blood glucose levels (r = -0.22, p = 0.06, n = 73). Changes in TT did not correlate with changes in waist circumference, systolic blood pressure, or SHBG levels.

## Discussion

Studies have observed a relationship between low testosterone and the components of metabolic syndrome [21], which would suggest that a hypogonadal population would have an increased prevalence of MetS. However, in the TRiUS population of hypogonadal men, 37% of the evaluated patients met the criteria for metabolic syndrome at baseline, which is consistent with the prevalence of metabolic syndrome in US adult men (34.8%-41.9%) [22]. We did find that low TT levels (<206 ng/dL [<7.1 nmol/L]) increased the risk of being classified as MetS+ and that TT levels significantly correlated with some MetS components before and after TRT. We found that TRT significantly increased testosterone levels in both MetS+ and MetS- hypogonadal populations and both achieved similar TT levels. Changes in MetS components, however, were only seen in MetS+ patients, including decreased fasting blood glucose levels, waist circumference, and BP. Although these changes were significant, no MetS+ patients changed MetS status. Given the patients' high baseline measurements, the need to have improvement across 1-3 components concurrently to change status, and the fact that the mean improvements did not break through the lower thresholds for MetS criteria, this may not be surprising.

In the literature, there is robust evidence of an association between metabolic conditions and low testosterone and/or SHBG [21,23-26]. Low testosterone levels are associated with, and predictive of, weight gain [27], central obesity [8,28], hypertension [29], MetS [8], insulin resistance [30], and type 2 diabetes [9,24,30,31]. Low testosterone is found in 33% of type 2 diabetes patients, including those younger than 35 years, as compared to only 6% in men with type 1 diabetes [26]. In prostate cancer patients who undergo chemical castration therapy, testosterone levels are suddenly and drastically reduced, resulting in increased fat deposition, increased insulin levels, impaired insulin sensitivity, and increased risk of diabetes and MetS classification [32,33]. The evidence for the association between hypogonadism and diabetes is strong enough to warrant its inclusion in the most current treatment guidelines for hypogonadism [2], which recommend screening for low testosterone in patients with type 2 diabetes. Our findings that TT levels correlated with waist circumference and fasting blood glucose in a hypogonadal population are in line with these observations.

However, it is unclear to what extent TRT can improve metabolic symptoms in hypogonadal men. Studies of TRT in hypogonadal men have shown improvements in body composition (fat-to-lean mass ratio) [11] and insulin sensitivity that can reliably be seen early in TRT (1-6 weeks) [34]. Changes in BMI are not always seen since a loss of fat weight can be replaced by lean muscle weight after TRT [35]. Recently, in a hypogonadal male population with type 2 diabetes and/or metabolic syndrome, Jones and colleagues [17] showed a 15.2% reduction in HOMA-IR (homeostasis model of assessment--insulin resistance) compared to placebo after 6 months of TRT use. This was maintained through the 12 months of the study. We found that TRT resulted in lowered waist circumference (visceral fat) and fasting blood glucose, but only in patients with very low testosterone levels (<206 ng/dL [<7.1 nmol/L]) or in MetS+ patients. We saw neither improvement, nor worsening, in lipid parameters in either MetS+ or MetS- cohorts; this reflects the results reported in a review by Jones [34], which found that the effect of TRT on HDL cholesterol is reduction in 25% of the studies, increase in 25% of the studies, and no effect in 50% of the studies.

In the MetS+ population of our study, improvements were generally seen at later time points: at 12 months for waist circumference and fasting blood glucose levels, and at 6 months for BP. This generally agrees with the literature, and it is possible that metabolic benefits of TRT may not be seen until later time points. At 3 months TRT, placebo-controlled studies in men with type 2 diabetes demonstrated mixed results; there were significant reductions in waist circumference but not BMI [13], reductions in waist circumference and fasting blood glucose, but not BP [12], and reductions in body weight and blood glucose [36]. In a longer, 8-month placebo-controlled trial of TRT in hypogonadal obese men, improvements over placebo were seen in visceral (abdominal) fat mass, blood glucose, diastolic BP, and serum cholesterol, but not BMI [37]. In a 12-month study of hypogonadal men with MetS, improvements were seen at 6 months in insulin sensitivity, waist circumference, and fat mass, and further improvements were seen at 12 months [15]. In a 2-year study by Haider et al of hypogonadal men treated with parenteral testosterone undecanoate, BMI, waist size, and weight continued to decrease over the full 24 months, while improvements in fasting glucose and lipid levels were seen only during the first 12 months [38]. It should be noted that in this study, 77% (37/47) of men with MetS no longer met the criteria for the condition after receiving TRT for 2 years [38].

In addition to prolonged use, metabolic benefit has also been seen when TRT is used in combination with weight loss. Heufelder et al conducted a 12-month, placebo controlled study of diet and exercise with or without TRT in 32 hypogonadal men with MetS and newly diagnosed type 2 diabetes [14]. They found that while there were improvements in all MetS components in both groups, addition of TRT to diet and exercise significantly improved glycemic control and waist circumference over diet and exercise alone. Using the ATP III definition of MetS as we did in the current study, they found that 81.3% of the TRT+diet+exercise group and 31.3% of the diet+exercise group no longer met the criteria for MetS after 12 months in the study. Our patient registry showed that TRT alone in MetS+ patients can improve two of the five criteria for MetS (ie, decreased fasting blood glucose levels and waist circumference). This result may be more realistic in clinical practice since many patients struggle with diet and exercise regimens outside of a structured clinical study. Heufelder et al also found a small improvement in TT levels with diet and exercise alone, emphasizing the bidirectional link between testosterone and MetS criteria. Because we did not record changes in diet and exercise in our study, it is unknown if improved MetS criteria had any impact on testosterone levels in our study.

Importantly for prediabetic men, we found that TRT improved fasting glucose levels in MetS+ men. This relationship between testosterone and insulin was highlighted in Yialamas et al [32], who found decreased insulin sensitivity and increased fasting blood glucose levels after withdrawal of TRT in young, healthy men with idiopathic hypogonadotropic hypogonadism. Similar results have been reported by Pitteloud et al [39,40], though Rabiee et al [41] found no change in insulin sensitivity in response to serum testosterone suppression in healthy non-hypogonadal men. Thus, insulin sensitivity may be related to a chronic hypogonadal state, though the mechanism for this is currently unclear.

An important limitation of our study was the lack of data regarding concomitant medication use for diabetes, dyslipidemia, or hypertension; because the registry was not designed specifically to evaluate metabolic syndrome, this information was not systematically collected. Without consistent documentation of the use of these concomitant medications, it is not possible to accurately determine the extent to which the effects on metabolic parameters reflect effects of TRT, or an optimization of medications these patients may be using. Other limitations of our study were typical of an observational study or patient registry. Patient behavior and physician protocol and practice were not defined by the registry protocol. Thus, we observed a high degree of variability due to inconsistency in patient follow-up visits, hypogonadism defined at the physician's discretion, no testosterone washout period before enrollment, no placebo control, and no centralized laboratory testing facility. This last limitation may have contributed to the inconsistent change we observed in free testosterone after TRT; it is unclear why free testosterone did not correlate with TT, although it should be noted that a uniform method of free testosterone assessment was not stipulated in the registry (ie, calculated vs measured; analog assay vs mass spectrometry). Because our data reflect the natural behavior of patients and physicians, the findings can be generalized to patients seeking medical attention for hypogonadal symptoms and those being treated for hypogonadism.

## Conclusions

Our findings are consistent with the literature showing an association between TT levels and MetS and the positive influence of TRT on waist circumference and fasting blood glucose levels. Furthermore, we show that 6 to 12 months of TRT may be required for more benefit in the typical hypogonadal patient with MetS. Numerous small studies have provided qualitative evidence that TRT provides metabolic benefit. In addition to the recently published results from the TIMES2 study [17], there is a need for large, long-term, placebo-controlled trials confirming the important observed benefits of TRT in hypogonadal men with MetS. In clinical practice, testing for low testosterone may be warranted in men with MetS who also exhibit other signs or symptoms of testosterone deficiency.

## Competing interests

**RKB **- Amgen: speaker; Auxilium: consultant, steering committee; Bristol-Myers Squibb: speaker; Novartis: speaker; Solvay: speaker. **MK **- Allergan: research funding; Auxilium: speaker; Boehringer Ingelheim: speaker; Slate: speaker. **GB **- Auxilium: investigator, speaker; Tibotec: advisor/consultant, speaker; ViiV: advisor/consultant, speaker; Virxsys: research funding, advisor/consultant. **HK **- Auxilium: employee. **DN **- Auxilium: employee. **MMM **- Auxilium: research funding; advisor; GlaxoSmithKline: research funding; Endo: advisor.

## Authors' contributions

All authors contributed equally and each were involved in study design, data acquisition, or data analysis/interpretation and in drafting or critically revising the manuscript. All authors reviewed the final manuscript and gave approval for publication.

## Prior presentation/publication

Data from this paper were presented at the Endocrine Society 2010 Annual Meeting (June 19-22, 2010, San Diego, California) and published in abstract form as Bhattacharya RK, et al. *Endocrine Reviews*. 2010;31(3 suppl 1):S466. Abstract P1-399. A companion paper describing the methodology and baseline symptoms and comorbidities of participants in the TRiUS registry has been published: Miner MM, Khera M, Bhattacharya R, Blick G, Kushner H. Baseline data from the TRiUS registry: symptoms and comorbidities of testosterone deficiency. *Postgrad Med*. 2011;123:17-27.

## Pre-publication history

The pre-publication history for this paper can be accessed here:

http://www.biomedcentral.com/1472-6823/11/18/prepub
